# Preoperative diagnosis and laparoscopic management of Amyand's hernia with isolated factor VII deficiency: A case report

**DOI:** 10.1016/j.ijscr.2025.111934

**Published:** 2025-09-12

**Authors:** Abdulkaream Bajafar, Rawan Mohamed, Malaz Elaagib, Mohamed Ali

**Affiliations:** a Department of General Surgery, East Jeddah Hospital, Jeddah, Saudi Arabia

**Keywords:** Laparoscopic hernioplasty, Coagulation abnormality, Inguinal mass, Haemostatic therapy, Perioperative care, Case report

## Abstract

**Introduction and importance:**

Amyand's hernia is a rare condition in which the vermiform appendix herniates through the inguinal canal, with an estimated prevalence of 1 %. Isolated factor VII deficiency is part of a rare group of coagulopathies, with an incidence of 1 in 50,000. To our knowledge, this is the first documented report of Amyand's hernia with isolated factor VII deficiency**.**

**Case presentation:**

We present a 59-year-old male with a 4-month history of progressive right scrotal swelling. Computed tomography revealed herniation of the appendix into the inguinal canal, confirming Amyand's hernia. Routine preoperative laboratory evaluation showed factor VII deficiency. Transabdominal preperitoneal hernia (TAPP) repair was done successfully without appendectomy, as the appendix was not inflamed.

**Clinical discussion:**

Amyand's hernia is often diagnosed intraoperatively, though CT and ultrasonography enable preoperative recognition. Management is typically guided by the Losanoff and Basson classification. The role of prophylactic appendectomy in uninflamed appendices remains debated; in this case it was avoided to preserve a clean surgical field. The factor VII deficiency required perioperative recombinant factor VII replacement, which was successful.

**Conclusion:**

Amyand's hernia with isolated factor VII deficiency is rare and has no standard treatment protocol, but we were able to perform surgery safely with appropriate precautions.

## Highlights

• First reported case of Amyand's hernia in a factor VII deficiency patient
• CT imaging enabled preoperative diagnosis of a rare inguinal hernia variant.
• Laparoscopic TAPP repair performed safely without appendectomy.
• Recombinant factor VIIa ensured haemostatic control during surgery.
• Coagulation screening revealed unexpected factor VII deficiency before surgery.

## 1 Introduction

Inguinal hernia is a condition in which contents of the abdominal cavity bulge through the inguinal canal either due to weakness in the canal's posterior wall (direct) or through the deep inguinal ring (indirect). It is a frequently encountered condition in the surgical field, with a reported lifetime risk of 27 % in men and 3 % in women [1]. Amyand's hernia is a rare entity of inguinal hernias in which the vermiform appendix protrudes through the hernia sac. Its prevalence is estimated to be less than 1 % [2], and its presentation can vary from asymptomatic to acute appendicitis.

The term Amyand's hernia is credited to Claudius Amyand, a French-born military surgeon sent to exile in England. Amyand performed the first ever reported successful appendectomy in an 11-year-old boy, Hanvil Anderson, in 1735 [3]. Whilst inguinal hernias can readily be diagnosed via simple physical examination, the diagnosis of Amyand's hernia, even with thorough clinical examination, is challenging, and most cases are incidentally discovered intraoperatively. However, with the advent of imaging techniques such as CT imaging and ultrasonography, the preoperative diagnosis of Amyand's hernia is becoming more feasible, thereby enhancing the utility of such diagnosis to the surgeon [4].

We present the case of a patient with Amyand's hernia diagnosed preoperatively using CT imaging. The preoperative evaluation indicated that he has a concomitant factor VII deficiency, which is an uncommon clotting condition in and of itself. This further adds to the case's peculiarity. The dual rarity of this patient's presentation emphasizes how important it is for clinicians to have a customized approach when dealing with such cases.

This case report has been reported in line with the SCARE checklist [5].

## 2 Case presentation

### 2.1 Clinical examination and initial evaluation

A 59-year-old male with an unremarkable medical history visited the General Surgery outpatient clinic complaining of right inguinal swelling. The swelling has been present for 4 months, with progressively increasing pain exacerbated by physical effort.

He reported a remote history of weightlifting dating decades back and a positive family history of inguinal hernias in several family members, including his son, brother and uncle.

Physical examination revealed a cooperative man who was alert and not in acute distress. Abdominal examination revealed a soft and reducible right inguinal swelling with a positive cough impulse. There were no signs of strangulation, and the overlying skin was normal. Examination of the contralateral side showed no observable pathology. At the time, in our setting, a CT scan was more readily available than ultrasonography, so we proceeded with CT imaging to expedite the patient's evaluation.

### 2.2 Imaging findings

**Fig. 1.1:**
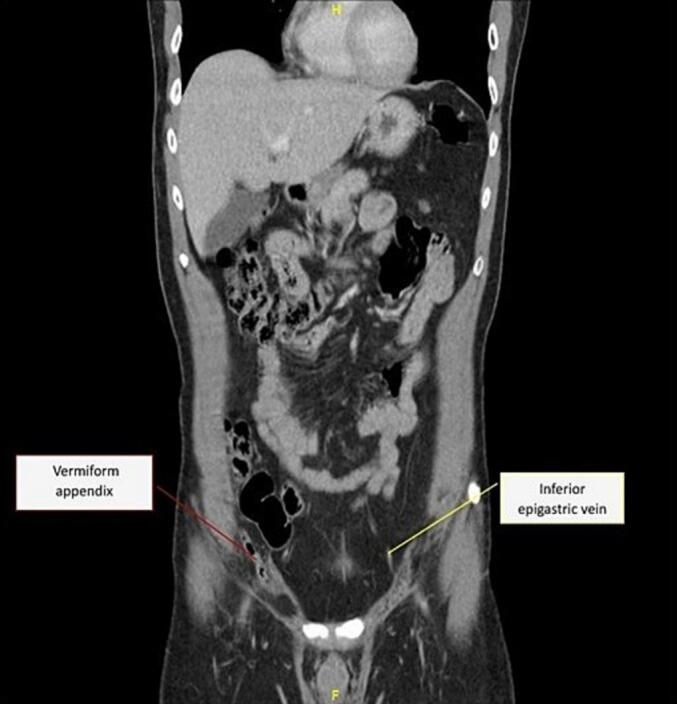
CT abdomen coronal view showing herniated appendix (red label). (For interpretation of the references to color in this figure legend, the reader is referred to the web version of this article.)

CT of the abdomen and pelvis confirmed a right inguinal hernia containing fat and the tip of the appendix. The hernial sack neck measured around 1 cm, and the appendix measured approximately 0.6 cm. There were also incidental findings of butterfly vertebrae at T10 and a possible splenic cyst, though these were deemed clinically insignificant (Fig. 1.1). A radiological diagnosis of Amyand hernia was made, and a plan for elective surgical repair was in order.

### 2.3 Preoperative workup

Subsequent preoperative laboratory investigations revealed a prolonged prothrombin time of 20.9 and an INR of 1.63. Factor VII activity was measured preoperatively and found to be 38 % (laboratory reference range: 55–177 %), confirming the diagnosis of isolated factor VII deficiency. The hematology team recommended administration of recombinant activated factor VII 3 mg intravenously 2 h before surgery and again 2 h postoperatively. The patient remained asymptomatic following surgery and has not attended hematology outpatient follow-up, in line with hematology advice, as no further intervention was deemed necessary. He was advised to inform treating physicians of his diagnosis before undergoing any future surgical or invasive procedures.

### 2.4 Surgical management and outcome

**Fig. 1.2:**
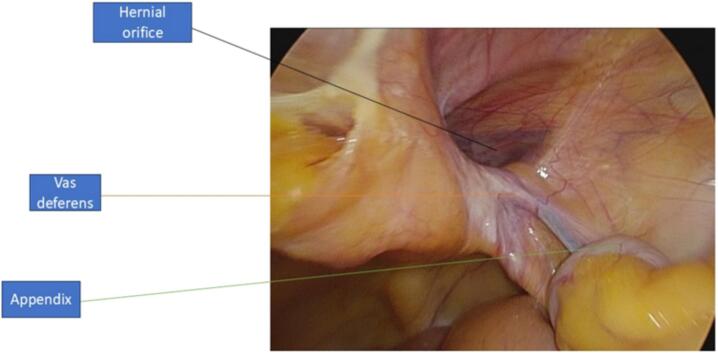
(TAPP repair).

**Fig. 1.3:**
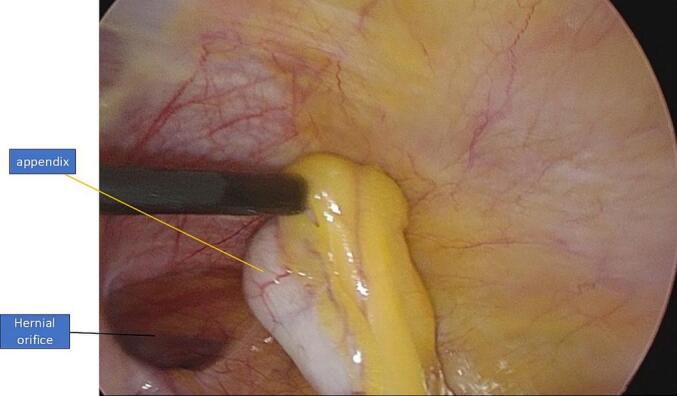
Uninflamed appendix (yellow label) reduced during TAPP repair. (For interpretation of the references to color in this figure legend, the reader is referred to the web version of this article.)

The surgical team performed a transabdominal preperitoneal repair (TAPP) to allow for thorough visualization of the hernial sac contents. A standard approach was used, and upon exploration, the appendix was identified within the hernial sac. The decision to perform an appendectomy or to use mesh repair is dynamic and often determined intraoperatively by the surgeon, taking into account factors such as the condition of the appendix, patient demographics, and comorbidities. Fortunately, the appendix appeared normal with no signs of inflammation, and it was readily reduced into the peritoneal cavity via gentle grasping with laparoscopic instruments. A pre-contoured (3D) mesh was not available; therefore, a flat self-gripping polypropylene mesh was used and trimmed intraoperatively to fit the defect (mediolateral dimension 15 cm, craniocaudal dimension 12 cm, and an 8 cm inferior extension), ensuring complete coverage of the myopectineal orifice with at least 3–4 cm overlap in all directions. The mesh was used to reinforce the inguinal floor and was secured in place using laparoscopic tackers. The contralateral side was also explored, and no additional hernia defects were found. There was no unusual bleeding during the operation. Operative time was approximately 90 min, which is consistent with standard TAPP repair duration (see Fig. 1.2, Fig. 1.3).

The patient's immediate postoperative course was uneventful without any complications, and he was discharged home on the first postoperative day. Over the ensuing three months, during follow-up in the outpatient clinic with the operating surgeon, the patient reported excellent symptomatic relief and was generally satisfied with the outcome.

## 3 Discussion

Amyand's hernia, though rare, presents the general surgeon with two frequently encountered conditions: inguinal hernias and appendicitis. Its rarity limits accurate prevalence estimates, which appear to be between 0.4 % and 0.6 %. Appendicitis occurring in association with Amyand's hernia is even rarer, with a reported prevalence of 0.1 % [6]. Other rare types of hernias include Littre's hernia (protrusion of a Meckel diverticulum), Richter's hernia (protrusion of a portion of the bowel wall), and De Garengeot's hernia (presence of the vermiform appendix within a femoral hernia).

Multiple theories have been proposed regarding the pathophysiology of Amyand's hernia. The most plausible hypothesis involves a patent processus vaginalis and a fibrous connection between the vermiform appendix and the testis, which guides the appendix through the inguinal canal. This hypothesis is supported by the observation that Amyand's hernia is three times more common in the pediatric population [6].

A question arises as to whether Amyand's hernia itself is a risk factor for appendicitis. It is reasonable to infer that an ischemic insult at the hernial sac could trigger inflammation within the appendix; however, this idea remains hypothetical, and more extensive studies are needed to confirm the proposed pathophysiology [3].

Most cases of Amyand's hernia are diagnosed intraoperatively, and routine sonographic or radiological assessments are not typically performed for uncomplicated inguinal hernias. However, the increasing feasibility of ultrasonography and computed tomography has led to more reports of preoperative diagnosis, as demonstrated in our case. Luchs et al. were the first to diagnose Amyand's hernia via CT, and Afkirat was the first to do so via ultrasonography [6]. On CT imaging, Amyand's hernia appears as a blind-ended tube arising from the cecum and passing through the inguinal canal.

In 2007, Losanoff and Basson proposed a classification system for managing Amyand's hernia, dividing it into four types based on the condition of the appendix [7] (Table 1). There is a consensus that if the appendix is inflamed, an appendectomy should be performed. However, debate persists regarding prophylactic appendectomy when the appendix appears normal. Proponents argue that the appendix is prone to herniation and may become inflamed later, particularly in younger patients [8]. In our case, we opted not to perform an appendectomy to avoid contaminating an otherwise clean surgical field and unnecessarily increasing the risk of infection; a standard TAPP repair with mesh was performed.

**Table 1:** Losanoff and Basson Classification of Amyand's Hernia.

Type	Description	Recommended Surgical Management
Type 1	Normal appendix within the inguinal hernia sac	Hernia reduction and mesh repair; appendectomy is not indicated
Type 2	Acute appendicitis within the hernia sac, without signs of abdominal sepsis	Appendectomy through the hernia sac followed by primary hernia repair without mesh
Type 3	Acute appendicitis with associated peritonitis or abdominal wall sepsis	Laparotomy, appendectomy, and hernia repair without mesh
Type 4	Amyand's hernia with acute appendicitis and unrelated intra-abdominal pathology	Management tailored according to Type 1–3 along with treatment of the additional intra-abdominal pathology

Interestingly, our patient's preoperative evaluation revealed an isolated factor VII deficiency. Factor VII deficiency is the most frequently encountered among rare bleeding disorders, with an incidence of 1 in 50,000 [9]. Its clinical presentation varies from severe bleeding to mild symptoms and does not necessarily correlate with the degree of deficiency. Although there are no previous reports on treating Amyand's hernia in a patient with isolated factor VII deficiency, the literature supports the safe use of recombinant factor VII infusion for hemostatic control in the perioperative setting [9]. Similar management was implemented in accordance with hematology recommendations, resulting in no immediate intra- or postoperative complications.

## 4 Conclusion

Amyand's hernia with isolated factor VII deficiency is rare and has no standard treatment protocol, but we were able to perform surgery safely with appropriate precautions.

## CRediT authorship contribution statement

-Abdulkaream Bajafar: Served as the Most Responsible Physician (MRP) for the case, supervised the project, contributed to the manuscript content, and critically reviewed it for intellectual and clinical accuracy.

-Rawan Mohamed: performed a literature review, and drafted portions of the manuscript.

-Malaz Elaagib: Contributed to editing and refining the manuscript, assisted in organizing the structure and discussion, and provided input on final formatting.

-Mohamed Ali: Participated in data acquisition, assisted in manuscript writing, and contributed to the integration of clinical details and references.

## Consent

Written informed consent was obtained from the patient for publication and any accompanying images. A copy of the written consent is available for review by the Editor-in-Chief of this journal on request**.**

## Ethical approval

Ethical review and approval were not required for the case report, as our institution waives such approval. The case report doesn't contain any personal information about the patient.

## Guarantor

Abdulkaream Bajafar.

## Registration of research studies

Not applicable.

## Funding

This research did not receive any specific grant from funding agencies in the public, commercial, or not-for-profit sectors.

## Name of the institution

East Jeddah General Hospital.

## Declaration of competing interest

The authors declare that they have no conflict of interest**.**
